# Chemopreventive Potential of Green Tea Catechins in Hepatocellular Carcinoma

**DOI:** 10.3390/ijms16036124

**Published:** 2015-03-17

**Authors:** Masahito Shimizu, Yohei Shirakami, Hiroyasu Sakai, Masaya Kubota, Takahiro Kochi, Takayasu Ideta, Tsuneyuki Miyazaki, Hisataka Moriwaki

**Affiliations:** Department of Medicine/Gastroenterology, Gifu University Graduate School of Medicine, 1-1 Yanagido, Gifu 501-1194, Japan; E-Mails: shirakamiyy@yahoo.co.jp (Y.S.); sakaih03@gifu-u.ac.jp (H.S.); kubota-gif@umin.ac.jp (M.K.); kottii924@yahoo.co.jp (T.K.); taka.mailbox.789@gmail.com (T.I.); tsunemiyazaking@yahoo.co.jp (T.M.); hmori@gifu-u.ac.jp (H.M.)

**Keywords:** chemoprevention, green tea catechins (GTCs), hepatocellular carcinoma (HCC), metabolic syndrome

## Abstract

Hepatocellular carcinoma (HCC), which is a common malignancy worldwide, usually develops in a cirrhotic liver due to hepatitis virus infection. Metabolic syndrome, which is frequently complicated by obesity and diabetes mellitus, is also a critical risk factor for liver carcinogenesis. Green tea catechins (GTCs) may possess potent anticancer and chemopreventive properties for a number of different malignancies, including liver cancer. Antioxidant and anti-inflammatory activities are key mechanisms through which GTCs prevent the development of neoplasms, and they also exert cancer chemopreventive effects by modulating several signaling transduction and metabolic pathways. Furthermore, GTCs are considered to be useful for the prevention of obesity- and metabolic syndrome-related carcinogenesis by improving metabolic disorders. Several interventional trials in humans have shown that GTCs may ameliorate metabolic abnormalities and prevent the development of precancerous lesions. The purpose of this article is to review the key mechanisms by which GTCs exert chemopreventive effects in liver carcinogenesis, focusing especially on their ability to inhibit receptor tyrosine kinases and improve metabolic abnormalities. We also review the evidence for GTCs acting to prevent metabolic syndrome-associated liver carcinogenesis.

## 1. Introduction

Hepatocellular carcinoma (HCC) is one of the most common malignancies and results in high mortality; an estimated 748,300 new cases and 695,900 deaths occur worldwide in a single year as a result [1]. HCC usually develops in the livers of patients with chronic hepatitis and liver cirrhosis caused by persistent infection with hepatitis viruses, indicating that these patients are at high risk of liver carcinogenesis [2,3]. In addition to hepatitis virus infection, metabolic syndrome, which is strongly associated with obesity and diabetes mellitus, has been identified as a major risk factor for the development of HCC [4,5,6]. These findings strongly suggest that cirrhotic patients, especially those who are obese and have diabetes mellitus, are at high risk of developing HCC and, therefore, may be good candidates for chemoprevention strategies [7,8,9].

Tea, produced from dried leaves of the plant *Camellia sinensis*, is one of the most popular beverages worldwide. The possible beneficial effects of tea polyphenols (catechins), including cancer chemoprevention in humans, have been extensively investigated [10,11,12]. Among tea catechins, green tea catechins (GTCs) have been most extensively studied for their cancer chemopreventive and anti-cancer properties. In addition, the ability of GTCs to improve metabolic abnormalities and reduce body weight has been reported by a number of basic and clinical studies [13,14,15]. These reports indicate that GTCs, which can exert both chemopreventive and anti-metabolic syndrome effects, are promising agents to prevent the development of HCC, especially in obese liver cirrhotic patients with metabolic disorders.

In this article, we provide an overview of the clinical characteristics and molecular pathogenesis of HCC, focusing on the role of obesity and its related metabolic abnormalities. We also review the evidence that GTCs might prevent obesity- and metabolic syndrome-related liver carcinogenesis.

## 2. Clinical Characteristics and Molecular Pathogenesis of HCC

As described above, HCC development is frequently associated with chronic inflammation and subsequent cirrhosis of the liver induced by hepatitis virus infection. The annual rate of HCC development is approximately 7% in cirrhotic patients, and the recurrence rate for this malignancy 5 years after curative treatment may exceed 70% [16,17,18]. The high incidence of HCC development in the cirrhotic liver is associated with its characteristic mode of carcinogenesis, *i.e.*, “multicentric carcinogenesis” [19,20]. Liver cirrhosis is caused by continuous inflammation leading to severe fibrosis, and is regarded as a precancerous field that contains multiple, independent premalignant lesions (clones). As a result, curative treatment of HCC is extremely difficult once these clones or HCC develop in the cirrhotic liver. This is one of the reasons why the prognosis of patients with cirrhosis and HCC is poor; therefore, there is an urgent need to develop more effective strategies for the chemoprevention of HCC.

Like other cancers, HCC development is significantly associated with the accumulation of genetic alterations, such as mutations in the *p53* tumor suppressor gene and the *CTNNB1* gene that encodes β-catenin [21,22,23]. Changes in several cell signaling and metabolic pathways also play a key role in liver carcinogenesis. In particular, abnormalities in the expression and function of receptor tyrosine kinases (RTKs) and their downstream signaling pathways, including the Ras/extracellular signal-regulated kinase (ERK) and phosphoinositide 3-kinase (PI3K)/Akt signaling pathways, are critically involved in the proliferation of HCC cells and tumor progression [24,25]. Among these pathways, the insulin-like growth factor-1 (IGF-1)/IGF-1 receptor (IGF-1R) axis, which is one of the most commonly deregulated signaling pathways contributing to the development of several types of human malignancies including HCC [25,26], seems to be particularly interesting because this axis plays a key role in metabolic syndrome-related liver carcinogenesis [7,8,9], as discussed in the next section.

## 3. Obesity, Metabolic Syndrome, and HCC

Obesity, a serious healthcare problem worldwide, and its related metabolic disorders, including diabetes mellitus and insulin resistance, which are collectively known as “metabolic syndrome” have been recognized as major risk factors for the development of HCC [4,5,6]. Several meta-analyses have shown a significant association between the risk of liver carcinogenesis and obesity- and diabetes mellitus-related complications [27,28,29]. Furthermore, a higher body mass index and diabetes mellitus have been shown to increase the risk of HCC in patients with decompensated cirrhosis [5]. Insulin resistance and hyperleptinemia, both of which are frequently observed in obese individuals, also contribute to the increased risk of HCC recurrence after curative treatment [6,30]. Nonalcoholic fatty liver disease (NAFLD) is commonly associated with metabolic syndrome and can progress to nonalcoholic steatohepatitis (NASH), which in turn leads to liver cirrhosis and HCC development [31,32].

**Figure 1:**
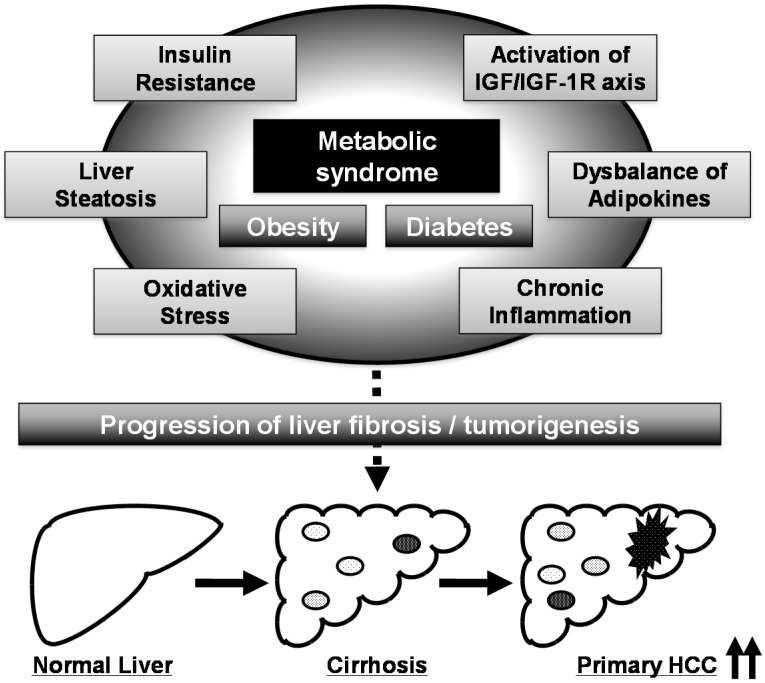
Proposed mechanisms linking metabolic syndrome and liver carcinogenesis. The black arrows at the right corner mean the incidence of hepatocellular carcinoma (HCC) is up-regulated.

Several pathophysiological mechanisms, including the emergence of insulin resistance, activation of the IGF/IGF-1R axis, development of adipokine imbalance, chronic inflammation, and induction of oxidative stress, link metabolic syndrome and hepatocarcinogenesis (Figure 1) [7,8,9]. Among these metabolic disorders, activation of the IGF/IGF-1R axis, which is caused by insulin resistance and hyperinsulinemia, is considered to play a key role in liver carcinogenesis in patients with metabolic syndrome [7,8,9]. Obese patients have been shown to have higher circulating levels of IGF-1 than non-obese patients in the presence of hyperinsulinemia [33], and animal studies have revealed that insulin resistance and activation of the IGF/IGF-1R axis are implicated in obesity- and diabetes-related liver carcinogenesis [34,35]. IGF-1R is overexpressed *in vitro* and in animal models of HCC [26,36,37], suggesting that activation of the IGF/IGF-1R axis and its related signaling pathways, such as the PI3K/Akt signaling pathway, significantly contribute to the stimulation of HCC cell growth and liver carcinogenesis, especially in the presence of metabolic disorders.

## 4. HCC Chemoprevention by Targeting Metabolic Disorders Caused by Metabolic Syndrome

Because metabolic abnormalities caused by metabolic syndrome have a great impact on liver carcinogenesis, we considered the possibility that metabolic disorders, such as insulin resistance and activation of the IGF/IGF-1R axis, might be effective targets in the prevention of HCC, and we tested this using animal models of obesity- and diabetes-related liver carcinogenesis.

We found that supplementation with branched-chain amino acids (BCAA), which improve protein malnutrition in patients with liver cirrhosis [38], significantly inhibited liver carcinogen diethylnitrosamine (DEN)-induced hepatocarcinogenesis as well as spontaneously occurring hepatic preneoplastic lesions in *db*/*db* obese and diabetic mice [34,39]. In these studies, BCAA supplementation suppressed liver carcinogenesis by reducing insulin resistance, ameliorating hepatic steatosis, inhibiting activation of the IGF/IGF-1R axis, and attenuating hepatic inflammation [34,39]. BCAA significantly inhibited the proliferation of HCC cells induced by visfatin, an adipokine, through the induction of apoptosis and cell-cycle arrest in G_0_/G_1_ phase [40]. Administration of acyclic retinoid, which can suppress the post-therapeutic recurrence of HCC and improve the survival rate of patients [41,42], also inhibited DEN-induced liver tumorigenesis in *db*/*db* mice, and this was associated with improved liver steatosis and insulin sensitivity [43]. In addition, pitavastatin, a drug widely used for the treatment of hyperlipidemia, inhibited the early phase of obesity-related liver tumorigenesis by improving liver steatosis, increasing serum levels of adiponectin, and attenuating chronic inflammation induced by excess fat deposition in the liver of *db*/*db* mice [44]. The possible chemopreventive properties of GTCs in metabolic syndrome-related liver carcinogenesis have also been investigated, and are described later.

## 5. Preventive and Therapeutic Potential of GTCs in Metabolic Syndrome

Beneficial effects of GTCs on metabolic syndrome-related parameters, including decreased body weight and adipose mass, as well as improved glucose homeostasis and dyslipidemia, have been found in a number of preclinical animal studies (for more detail, see reviews [13,14]). Administration of (−)-epigallocatechin gallate (EGCG), a major component of GTCs, through drinking water significantly decreased the amount of white adipose tissue and serum triglyceride levels in *db*/*db* mice [35,45]. In cell culture studies, treatment with GTCs inhibited preadipocyte differentiation, decreased adipocyte proliferation, induced adipocyte apoptosis, and suppressed cellular triglyceride accumulation [13,14]. Moreover, several human studies found that green tea administration was effective at preventing metabolic syndrome and controlling body weight [13,14,15]. The results of a cross sectional study in a Japanese population showed that green tea consumption was significantly associated with lower circulating levels of aminotransferases, triglycerides, and atherogenic lipoproteins [46]. Meta-analyses by Hursel *et al.* [47,48] also revealed that GTCs combined with caffeine had a significant effect on weight loss, weight maintenance, and energy expenditure.

GTCs also exert preventive and therapeutic effects on pathological changes in the liver associated with metabolic syndrome [49]. Treatment with EGCG improved hepatic histology, such as fibrosis and steatohepatitis, and attenuated oxidative stress and inflammation in an experimental rat model of NAFLD/NASH [50,51,52]. In a double-blind placebo-controlled study, drinking green tea containing high-density catechins reduced the percentage of body fat and hepatic fat infiltration, improved liver function, and attenuated oxidative stress in patients with NAFLD [53]. These findings strongly suggest that supplementation with GTCs is a potentially viable strategy for the treatment of NAFLD and, probably, for the prevention of its progression to NASH.

## 6. Molecular Mechanisms for Anti-Cancer Effects of GTCs and Chemopreventive Potential in Liver Carcinogenesis

Chemopreventive and anti-cancer effects of GTCs in HCC have been reported in several *in vitro* studies (for more detail, see review [54]). EGCG inhibits the growth and proliferation of human HCC-derived cells by inducing apoptosis [55,56,57]. EGCG also suppresses the growth of HepG2 human HCC cells by inhibiting the phosphorylation of IGF-1R, followed by decreased activation of its downstream signaling molecules, including ERK, Akt, STAT-3, and GSK-3β [58]. In the literature, it is shown that the levels of both IGF-1 and IGF-2 in cells and culture media are decreased by treatment with EGCG, while the levels of IGF binding protein-3 are increased. In addition, treatment of HuH7 human HCC cells with EGCG decreases the expression of both phosphorylated and non-phosphorylated vascular endothelial growth factor (VEGF) receptor-2 (VEGFR-2) proteins [59]. EGCG also inhibits HuH7 cell proliferation and down-regulated the levels of VEGF in culture media, suggesting that EGCG may be able to inhibit cell growth by disrupting the VEGF-VEGFR related autocrine loop existing in HCC cells. These two article, focusing on IGF-1R and VEGFR-2, indicate that certain types of RTKs and their downstream pathways are key targets of GTCs and hence mediators of their chemopreventive and anti-cancer properties. Moreover, EGCG suppresses platelet-derived growth factor (PDGF)-induced cell proliferation in human hepatic stellate cells by inhibiting the phosphorylation of PDGF receptor (PDGFR), another RTK [60]. Along with the direct effects of EGCG on certain types of RTKs at the cell surface, several studies have revealed that EGCG has indirect effects on RTKs by targeting the lipid organization of the plasma membrane, lipid rafts. EGCG treatment alters the lipid rafts of cancer cells and inhibits binding of the ligand epidermal growth factor (EGF) to the EGF receptor (EGFR), which is an RTK as well, and the subsequent receptor dimerization [61]. EGCG also decreases cell surface EGFR by inducing the internalization of EGFR into endosomal vesicles, leading to inhibiting the activation of the receptor and exerting anti-cancer effects [62]. Although only colon cancer cells are employed in these examinations, the indirect effects on cell surface RTKs appear to be one of the putative mechanisms of GTCs against liver cancer. Administration of EGCG through drinking water also prevented carbon tetrachloride (CCl_4_)-induced rat hepatic fibrosis by inhibiting IGF-1R and PDGFR-β expression [63]. These findings may be particularly significant because, in addition to HCC cells, GTCs also target several types of RTKs in a variety of other cell types, and this might contribute to the prevention of liver fibrosis progression, a precancerous condition of HCC development.

A number of *in vivo* preclinical studies, including chemically induced models, tumor xenograft models, and spontaneous models, have found that GTCs have chemopreventive effects in liver carcinogenesis [54]. Supplementation with GTCs significantly suppressed the development of glutathione *S*-transferase placental form (GST-P)-positive (GST-P^+^) foci, a hepatic preneoplastic lesion, induced by DEN via reduction of oxidative stress [64]. The growth of HCC xenografts was suppressed by administration of EGCG in drinking water, and this was associated with the induction of apoptosis [56]. EGCG also suppressed the growth of HuH7 xenografts in nude mice by preventing activation of the VEGF/VEGFR signaling axis [59]. Furthermore, EGCG administration also reduced the incidence of spontaneous HCC development in mice [65]. The results of these preclinical studies demonstrate that GTCs can potentially prevent the early phase of liver carcinogenesis and inhibit the growth of existing HCC.

## 7. Chemopreventive Potential of GTCs in Metabolic Syndrome-Related Liver Carcinogenesis

In order to evaluate the potential of GTCs to act as chemopreventive agents for metabolic syndrome-related liver carcinogenesis, we have performed a number of animal studies [35,51,52]. In the first of these, we examined the effects of EGCG on the development of DEN-induced liver tumorigenesis in *db*/*db* mice. In this study, drinking EGCG significantly inhibited the development of liver cell adenomas compared to the control group (EGCG-untreated), and this was associated with reduced phosphorylation of IGF-1R, ERK, and Akt proteins in the liver, improvement of liver steatosis, and activation of AMP-activated kinase protein in the liver. Interestingly, the serum levels of insulin, IGF-1, IGF-2, free fatty acid, and tumor necrosis factor (TNF)-α, and the hepatic expression levels of *TNF-α*, *interleukin (IL)-6*, *IL-1β*, and *IL-18* mRNAs, were decreased after the administration of EGCG in drinking water [35].

Next, we investigated whether EGCG could inhibit liver tumorigenesis using a novel rat model that shows histopathological and pathophysiological characteristics of NASH. We found that the liver of Sprague-Dawley rats treated with DEN and a high-fat diet (HFD) exhibited marked development of GST-P^+^ foci associated with severe hepatic steatosis and inflammation, increased levels of oxidative stress, and enhancement of hepatocyte proliferation. EGCG administration in drinking water significantly inhibited the development of GST-P^+^ foci by reducing hepatic triglyceride content and hepatic fibrosis, lowering oxidative stress, attenuating inflammation, and inhibiting excessive hepatocyte proliferation in these rats [51].

**Figure 2:**
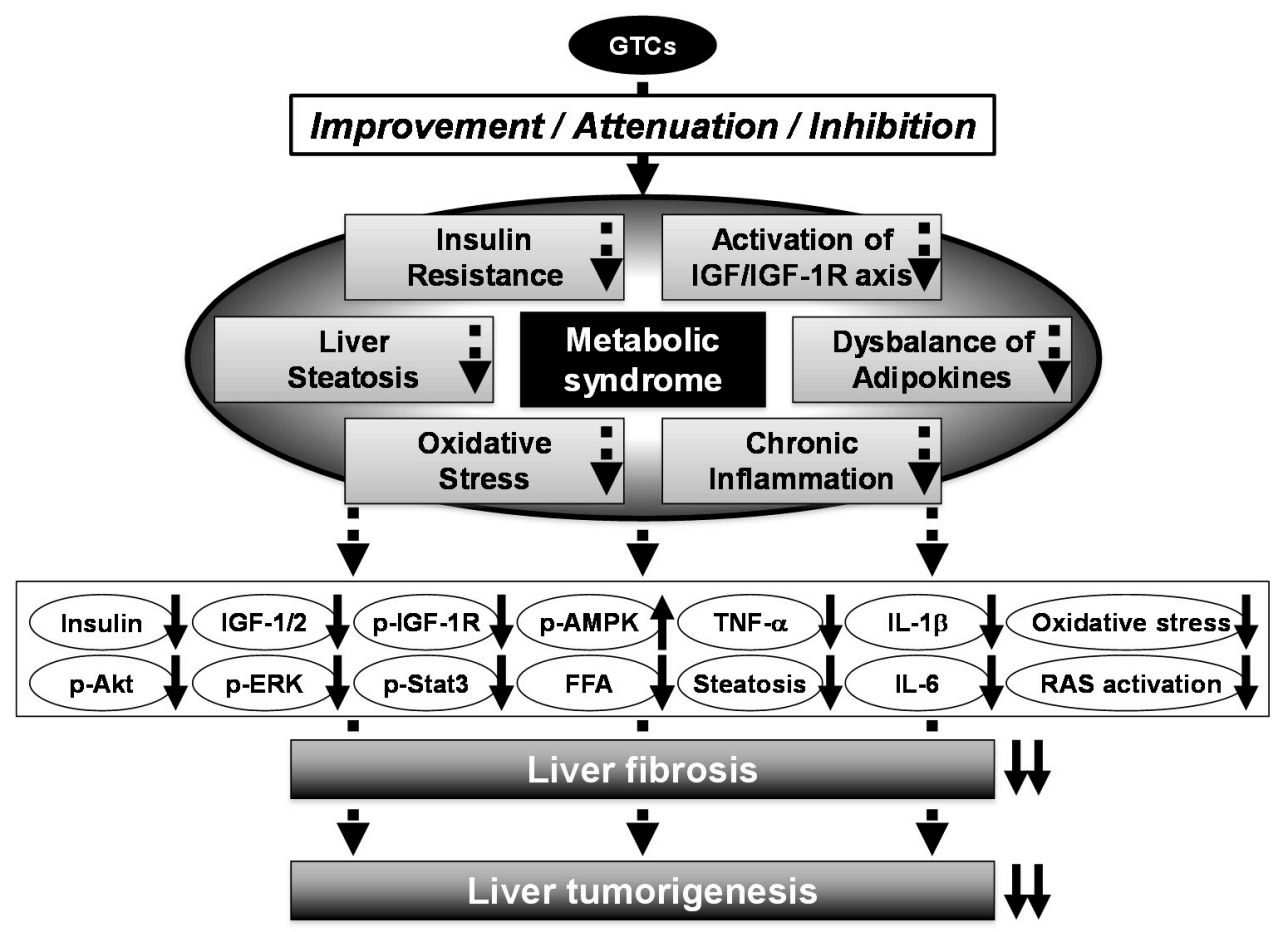
Mechanisms of action of GTCs in the inhibition of metabolic syndrome-related liver carcinogenesis. Metabolic syndrome significantly increases the risk of HCC development. Several pathophysiological mechanisms link metabolic syndrome and liver carcinogenesis, such as insulin resistance, activation of the IGF/IGF-1R axis, chronic inflammation, oxidative stress, and adipokine dysbalance. They appear to be followed by molecular abnormality, including activation of PI3K/Akt and MAPK/ERK signaling, down-regulated phosphorylated-AMPK, and increased pro-inflammatory cytokines. Administration of GTCs significantly reduces the risk of HCC development in obese patients, and this might be associated with decreased insulin resistance and hepatic steatosis, inhibition of the activation of the IGF/IGF-1R axis, and attenuation of oxidative stress and chronic inflammation. Up- and down-pointing solid arrows mean items are up- and down-regulated, respectively.

Recently, we have also developed a novel NASH-related liver tumorigenesis model using SHRSP.Z-*Lepr^fa^*/IzmDmcr (SHRSP-ZF) rats, which present with obesity, diabetes, and hypertension and thus mimic human metabolic syndrome; we examined whether EGCG inhibited the development of GST-P^+^ foci in these rats. When treated with CCl_4_ and HFD, SHRSP-ZF rats showed liver dysfunction, obesity, insulin resistance, dyslipidemia, adipokine imbalance in the serum, and hepatic steatosis significantly more frequently than control non-obese and normotensive rats. The development of GST-P^+^ foci and liver fibrosis was markedly accelerated in SHRSP-ZF rats compared to control rats, and this was associated with activation of the renin-angiotensin system (RAS) and induction of inflammation and oxidative stress. However, administration of EGCG in drinking water significantly suppressed the development of GST-P^+^ foci by improving liver fibrosis, inhibiting RAS activation, and attenuating inflammation and oxidative stress in CCl_4_- and HFD-treated SHRSP-ZF rats [52]. The results of these animal studies suggest that administering GTCs, which possess preventive and therapeutic properties against metabolic disorders, might serve as an effective chemoprevention modality for obesity- and metabolic syndrome-related liver tumorigenesis (Figure 2) [35,51,52].

## 8. Clinical Trial Using GTCs and Future Directions

Several series of preclinical investigations using both cell cultures and rodent models have strongly indicated that GTCs have chemopreventive and therapeutic potential in human cancers. In a pilot study, we found that, compared to an untreated control group, oral supplementation with green tea extract (1.5 g/day for 1 year) significantly reduced the development of metachronous colorectal adenomas, the precancerous lesions for colorectal cancer, after polypectomy [66]. The results from a randomized, double-blinded, placebo-controlled study showed that oral administration of GTCs for 1 year also inhibited the progression of high grade prostate intraepithelial neoplasia to prostate cancer [67]. Moreover, oral administration of mixed tea products significantly decreased the size of leukoplakia, a precancerous lesion of the oral mucosa [68]. These findings suggest that an interventional approach using GTCs may be effective in the prevention of certain types of tumorigenesis, especially in early stages.

It is believed that consumption of green tea in its whole form provides greater anti-cancer effect than supplementation with EGCG alone. Indeed, several reports have indicated that EGCG exerts more significant cancer chemopreventive effect when administered in combination with other tea catechins [69,70,71]. Our findings have also shown that EGCG treatment combined with (−)-epicatechin (EC) results in synergistic inhibition of cell growth in the HT29 human colon cancer cell line [72]. According to the combination assay, the optimal ratio of EGCG to EC for effective anti-cancer activity is 60:7. The ratio reflects the relative concentration of EGCG to EC of Polyphenon E (PolyE), which contains 65% EGCG, 25% other catechins, and 0.6% caffeine, extracted from green tea. PolyE is reported to display anti-cancer effects almost equivalent to that of the same concentration of EGCG alone in *in vitro* study [72]. Other studies in rodents have also demonstrated greater efficacy from PolyE in inhibiting tumor development compared to GTCs [10,73]. These findings suggest that the anti-cancer effect of GTC may differ depending on its composition or combination with other agents.

The safety of GTCs as a chemopreventive agent has been demonstrated in several studies; no adverse events are observed with administration of tea polyphenols at doses ranging from 600 to 1800 mg/day [66,67,74,75]. In human interventional studies, relatively high doses of GTCs seem to be often used to achieve high concentrations of catechins in the blood and tissues. Therefore, a number of case reports of side effects related to administration of GTCs can be seen; these include excess gas, nausea, heartburn, abdominal pain, dizziness, headache, muscle pain, and hepatotoxicity [75,76]. These adverse reactions appear to be experienced in examinations with supplementation of high doses of GTCs in pills or capsules rather than drinking green tea [77]. Recent literature indicates that green tea extract, including GTCs, may inhibit certain types of microsomal cytochrome P450 (CYP), and may not lead to drug-induced liver injury when a drug and green tea are administered simultaneously [78].

A randomized, double-blind, placebo-controlled phase IIa chemoprevention trial demonstrated that GTCs have antioxidant effects in individuals who have several risk factors for HCC, and this may suggest that chemoprevention with GTCs is an effective strategy for diminishing oxidative DNA damage [79]. Conversely, Jin *et al.* [80] reported that GTCs did not significantly reduce HCC incidence or HCC-related mortality in a review of four clinical studies. The authors found that there were major limitations that needed to be taken into account when interpreting the results of these studies, including an improper comparison of control and treatment groups, possible selection bias, and a lack of randomization. In addition, the dosage and duration of GTC administration varied between studies, and this might have affected the results of these studies. Therefore, large-scale, randomized, double-blinded, placebo-controlled studies should be conducted to clarify the chemopreventive effects of GTCs on HCC development in chronic liver disease patients, especially those who have metabolic syndrome or are obese. Interestingly, the serum levels of IGF-1, VEGF, and prostate-specific antigen were significantly decreased following the administration of a green tea mixture to prostate cancer patients [81]. Therefore, appropriate biomarkers, including HCC-related tumor markers and several types of growth factors associated with liver carcinogenesis and fibrosis, should be measured when evaluating the chemopreventive effects of GTCs on liver tumorigenesis in future clinical trials.

## 9. Concluding Remarks

Finally, we would like to discuss the interesting findings of our recent studies showing that specific agents may be able to prevent colorectal carcinogenesis associated with obesity and metabolic syndrome, both of which also increase the risk of colorectal cancer [82,83]. Specifically, we have reported that supplementation with both EGCG and BCAA suppresses obesity-related colorectal carcinogenesis in *db*/*db* mice by reducing serum levels of insulin and inhibiting activation of the IGF/IGF-1R signaling pathway in the colonic mucosa [45,84]. Supplementation with curcumin, a component of turmeric, also inhibits the development of chemically induced colonic premalignant lesions in *db*/*db* mice by attenuating chronic inflammation and reducing adipokine imbalance [85]. The results of these studies [45,84,85], together with the evidence summarized in this review, may indicate that a nutraceutical approach for targeting metabolic abnormalities could effectively prevent the development of certain types of cancers associated with obesity and metabolic syndrome [9,77,86]. In addition, as reviewed in this article, GTCs are one of the most practical agents to use with this treatment approach.

When considering current social and medical circumstances, there is a great concern that the number of patients with metabolic syndrome-associated HCC will continue to increase. GTCs are considered one of the most promising phytochemicals for the prevention of cancer and the treatment of metabolic disorders. From the present review, it is particularly noteworthy that the administration of GTCs is an effective strategy to prevent the development of metabolic syndrome-related liver tumorigenesis. In order to clarify the effectiveness of GTCs in suppressing metabolic syndrome-related liver tumorigenesis, further extensive study is needed, including an active intervention trial.

## Abbreviations

AMPK: adenosine monophosphate-activated protein kinase
BCAA: branched-chain amino acids
CCl_4_: carbon tetrachloride
CYP: cytochrome P450
DEN: *N*-diethylnitrosamine
EC: (−)-epicatechin
EGCG: (−)-epigallocatechin gallate
EGF: epidermal growth factor
EGFR: epidermal growth factor receptor
ERK: extracellular signal-regulated kinase
GSK: glycogen synthase kinase
GST-P: glutathione *S*-transferase placental form
GTCs: green tea catechins
HCC: hepatocellular carcinoma
HFD: high-fat diet
IGF-1: insulin-like growth factor-1
IGF-1R: insulin-like growth factor-1 receptor
IL-6: interleukin-6
MAPK: mitogen-activated protein kinase
NAFLD: non-alcoholic fatty liver disease
NASH: non-alcoholic steatohepatitis
PDGF: platelet-derived growth factor
PDGFR: platelet-derived growth factor receptor
PI3K: phosphoinositide 3-kinase
PolyE: Polyphenon E
RAS: renin-angiotensin system
RTKs: receptor tyrosine kinases
SHRSP-ZF: SHRSP.Z-*Lepr^fa^*/IzmDmcr
STAT: signal transducer and activator of transcription TNF-α, tumor necrosis factor-α
VEGF: vascular endothelial growth factor
VEGFR: vascular endothelial growth factor receptor

## References

[1] Jemal A., Bray F., Center M.M., Ferlay J., Ward E., Forman D. (2011). Global cancer statistics. CA Cancer J. Clin..

[2] El-Serag H.B., Rudolph K.L. (2007). Hepatocellular carcinoma: Epidemiology and molecular carcinogenesis. Gastroenterology.

[3] Parikh S., Hyman D. (2007). Hepatocellular cancer: A guide for the internist. Am. J. Med..

[4] El-Serag H.B., Tran T., Everhart J.E. (2004). Diabetes increases the risk of chronic liver disease and hepatocellular carcinoma. Gastroenterology.

[5] Muto Y., Sato S., Watanabe A., Moriwaki H., Suzuki K., Kato A., Kato M., Nakamura T., Higuchi K., Nishiguchi S. (2006). Overweight and obesity increase the risk for liver cancer in patients with liver cirrhosis and long-term oral supplementation with branched-chain amino acid granules inhibits liver carcinogenesis in heavier patients with liver cirrhosis. Hepatol. Res..

[6] Imai K., Takai K., Nishigaki Y., Shimizu S., Naiki T., Hayashi H., Uematsu T., Sugihara J., Tomita E., Shimizu M. (2010). Insulin resistance raises the risk for recurrence of stage I hepatocellular carcinoma after curative radiofrequency ablation in hepatitis C virus-positive patients: A prospective, case series study. Hepatol. Res..

[7] Shimizu M., Shirakami Y., Hanai T., Imai K., Suetsugu A., Takai K., Shiraki M., Moriwaki H. (2014). Pharmaceutical and nutraceutical approaches for preventing liver carcinogenesis: Chemoprevention of hepatocellular carcinoma using acyclic retinoid and branched-chain amino acids. Mol. Nutr. Food Res..

[8] Shimizu M., Tanaka T., Moriwaki H. (2013). Obesity and hepatocellular carcinoma: Targeting obesity-related inflammation for chemoprevention of liver carcinogenesis. Semin. Immunopathol..

[9] Shimizu M., Kubota M., Tanaka T., Moriwaki H. (2012). Nutraceutical approach for preventing obesity-related colorectal and liver carcinogenesis. Int. J. Mol. Sci..

[10] Yang C.S., Maliakal P., Meng X. (2002). Inhibition of carcinogenesis by tea. Annu. Rev. Pharmacol. Toxicol..

[11] Yang C.S., Wang X., Lu G., Picinich S.C. (2009). Cancer prevention by tea: Animal studies, molecular mechanisms and human relevance. Nat. Rev. Cancer.

[12] Shirakami Y., Shimizu M., Moriwaki H. (2012). Cancer chemoprevention with green tea catechins: From bench to bed. Curr. Drug Targets.

[13] Wang S., Moustaid-Moussa N., Chen L., Mo H., Shastri A., Su R., Bapat P., Kwun I., Shen C.L. (2014). Novel insights of dietary polyphenols and obesity. J. Nutr. Biochem..

[14] Sae-tan S., Grove K.A., Lambert J.D. (2011). Weight control and prevention of metabolic syndrome by green tea. Pharmacol. Res..

[15] Huang J., Wang Y., Xie Z., Zhou Y., Zhang Y., Wan X. (2014). The anti-obesity effects of green tea in human intervention and basic molecular studies. Eur. J. Clin. Nutr..

[16] Kumada T., Nakano S., Takeda I., Sugiyama K., Osada T., Kiriyama S., Sone Y., Toyoda H., Shimada S., Takahashi M. (1997). Patterns of recurrence after initial treatment in patients with small hepatocellular carcinoma. Hepatology.

[17] Koda M., Murawaki Y., Mitsuda A., Ohyama K., Horie Y., Suou T., Kawasaki H., Ikawa S. (2000). Predictive factors for intrahepatic recurrence after percutaneous ethanol injection therapy for small hepatocellular carcinoma. Cancer.

[18] Tsukuma H., Hiyama T., Tanaka S., Nakao M., Yabuuchi T., Kitamura T., Nakanishi K., Fujimoto I., Inoue A., Yamazaki H. (1993). Risk factors for hepatocellular carcinoma among patients with chronic liver disease. N. Engl. J. Med..

[19] Shimizu M., Takai K., Moriwaki H. (2009). Strategy and mechanism for the prevention of hepatocellular carcinoma: Phosphorylated retinoid X receptor α is a critical target for hepatocellular carcinoma chemoprevention. Cancer Sci..

[20] Shimizu M., Imai K., Takai K., Moriwaki H. (2012). Role of acyclic retinoid in the chemoprevention of hepatocellular carcinoma: Basic aspects, clinical applications, and future prospects. Curr. Cancer Drug Targets.

[21] Farazi P.A., DePinho R.A. (2006). Hepatocellular carcinoma pathogenesis: From genes to environment. Nat. Rev. Cancer.

[22] Villanueva A., Newell P., Chiang D.Y., Friedman S.L., Llovet J.M. (2007). Genomics and signaling pathways in hepatocellular carcinoma. Semin. Liver Dis..

[23] Tornesello M.L., Buonaguro L., Tatangelo F., Botti G., Izzo F., Buonaguro F.M. (2013). Mutations in *TP53*, *CTNNB1* and *PIK3CA* genes in hepatocellular carcinoma associated with hepatitis B and hepatitis C virus infections. Genomics.

[24] Berasain C., Avila M.A. (2014). The EGFR signalling system in the liver: From hepatoprotection to hepatocarcinogenesis. J. Gastroenterol..

[25] Enguita-German M., Fortes P. (2014). Targeting the insulin-like growth factor pathway in hepatocellular carcinoma. World J. Hepatol..

[26] Scharf J.G., Braulke T. (2003). The role of the IGF axis in hepatocarcinogenesis. Horm. Metab. Res..

[27] Larsson S.C., Wolk A. (2007). Overweight, obesity and risk of liver cancer: A meta-analysis of cohort studies. Br. J. Cancer.

[28] El-Serag H.B., Hampel H., Javadi F. (2006). The association between diabetes and hepatocellular carcinoma: A systematic review of epidemiologic evidence. Clin. Gastroenterol. Hepatol..

[29] Wang P., Kang D., Cao W., Wang Y., Liu Z. (2012). Diabetes mellitus and risk of hepatocellular carcinoma: A systematic review and meta-analysis. Diabetes Metab. Res. Rev..

[30] Watanabe N., Takai K., Imai K., Shimizu M., Naiki T., Nagaki M., Moriwaki H. (2011). Increased levels of serum leptin are a risk factor for the recurrence of stage I/II hepatocellular carcinoma after curative treatment. J. Clin. Biochem. Nutr..

[31] Angulo P. (2002). Nonalcoholic fatty liver disease. N. Engl. J. Med..

[32] Starley B.Q., Calcagno C.J., Harrison S.A. (2010). Nonalcoholic fatty liver disease and hepatocellular carcinoma: A weighty connection. Hepatology.

[33] Brick D.J., Gerweck A.V., Meenaghan E., Lawson E.A., Misra M., Fazeli P., Johnson W., Klibanski A., Miller K.K. (2010). Determinants of IGF1 and GH across the weight spectrum: From anorexia nervosa to obesity. Eur. J. Endocrinol..

[34] Iwasa J., Shimizu M., Shiraki M., Shirakami Y., Sakai H., Terakura Y., Takai K., Tsurumi H., Tanaka T., Moriwaki H. (2010). Dietary supplementation with branched-chain amino acids suppresses diethylnitrosamine-induced liver tumorigenesis in obese and diabetic C57BL/KsJ-*db*/*db* mice. Cancer Sci..

[35] Shimizu M., Sakai H., Shirakami Y., Yasuda Y., Kubota M., Terakura D., Baba A., Ohno T., Hara Y., Tanaka T. (2011). Preventive effects of (−)-epigallocatechin gallate on diethylnitrosamine-induced liver tumorigenesis in obese and diabetic C57BL/KsJ-*db*/*db* mice. Cancer Prev. Res. (Phila).

[36] Scharf J.G., Dombrowski F., Ramadori G. (2001). The IGF axis and hepatocarcinogenesis. Mol. Pathol..

[37] Tovar V., Alsinet C., Villanueva A., Hoshida Y., Chiang D.Y., Sole M., Thung S., Moyano S., Toffanin S., Minguez B. (2010). IGF activation in a molecular subclass of hepatocellular carcinoma and pre-clinical efficacy of IGF-1R blockage. J. Hepatol..

[38] Moriwaki H., Shiraki M., Fukushima H., Shimizu M., Iwasa J., Naiki T., Nagaki M. (2008). Long-term outcome of branched-chain amino acid treatment in patients with liver cirrhosis. Hepatol. Res..

[39] Terakura D., Shimizu M., Iwasa J., Baba A., Kochi T., Ohno T., Kubota M., Shirakami Y., Shiraki M., Takai K. (2012). Preventive effects of branched-chain amino acid supplementation on the spontaneous development of hepatic preneoplastic lesions in C57BL/KsJ-*db*/*db* obese mice. Carcinogenesis.

[40] Ninomiya S., Shimizu M., Imai K., Takai K., Shiraki M., Hara T., Tsurumi H., Ishizaki S., Moriwaki H. (2011). Possible role of visfatin in hepatoma progression and the effects of branched-chain amino acids on visfatin-induced proliferation in human hepatoma cells. Cancer Prev. Res. (Phila).

[41] Okita K., Izumi N., Matsui O., Tanaka K., Kaneko S., Moriwaki H., Ikeda K., Osaki Y., Numata K., Nakachi K. (2014). Peretinoin after curative therapy of hepatitis C-related hepatocellular carcinoma: A randomized double-blind placebo-controlled study. J. Gastroenterol..

[42] Okita K., Izumi N., Ikeda K., Osaki Y., Numata K., Ikeda M., Kokudo N., Imanaka K., Nishiguchi S., Kondo S. (2014). Survey of survival among patients with hepatitis C virus-related hepatocellular carcinoma treated with peretinoin, an acyclic retinoid, after the completion of a randomized, placebo-controlled trial. J. Gastroenterol..

[43] Shimizu M., Sakai H., Shirakami Y., Iwasa J., Yasuda Y., Kubota M., Takai K., Tsurumi H., Tanaka T., Moriwaki H. (2011). Acyclic retinoid inhibits diethylnitrosamine-induced liver tumorigenesis in obese and diabetic C57BLKS/J-+(*db*)/+Lepr(*db*) mice. Cancer Prev. Res. (Phila).

[44] Shimizu M., Yasuda Y., Sakai H., Kubota M., Terakura D., Baba A., Ohno T., Kochi T., Tsurumi H., Tanaka T. (2011). Pitavastatin suppresses diethylnitrosamine-induced liver preneoplasms in male C57BL/KsJ-*db*/*db* obese mice. BMC Cancer.

[45] Shimizu M., Shirakami Y., Sakai H., Adachi S., Hata K., Hirose Y., Tsurumi H., Tanaka T., Moriwaki H. (2008). (−)-Epigallocatechin gallate suppresses azoxymethane-induced colonic premalignant lesions in male C57BL/KsJ-*db*/*db* mice. Cancer Prev. Res. (Phila).

[46] Imai K., Nakachi K. (1995). Cross sectional study of effects of drinking green tea on cardiovascular and liver diseases. BMJ.

[47] Hursel R., Viechtbauer W., Westerterp-Plantenga M.S. (2009). The effects of green tea on weight loss and weight maintenance: A meta-analysis. Int. J. Obes. (Lond.).

[48] Hursel R., Viechtbauer W., Dulloo A.G., Tremblay A., Tappy L., Rumpler W., Westerterp-Plantenga M.S. (2011). The effects of catechin rich teas and caffeine on energy expenditure and fat oxidation: A meta-analysis. Obes. Rev..

[49] Masterjohn C., Bruno R.S. (2012). Therapeutic potential of green tea in nonalcoholic fatty liver disease. Nutr. Rev..

[50] Xiao J., Ho C.T., Liong E.C., Nanji A.A., Leung T.M., Lau T.Y., Fung M.L., Tipoe G.L. (2014). Epigallocatechin gallate attenuates fibrosis, oxidative stress, and inflammation in non-alcoholic fatty liver disease rat model through TGF/SMAD, PI3 K/Akt/FoxO1, and NF-κB pathways. Eur. J. Nutr..

[51] Sumi T., Shirakami Y., Shimizu M., Kochi T., Ohno T., Kubota M., Shiraki M., Tsurumi H., Tanaka T., Moriwaki H. (2013). (−)-Epigallocatechin-3-gallate suppresses hepatic preneoplastic lesions developed in a novel rat model of non-alcoholic steatohepatitis. SpringerPlus.

[52] Kochi T., Shimizu M., Terakura D., Baba A., Ohno T., Kubota M., Shirakami Y., Tsurumi H., Tanaka T., Moriwaki H. (2014). Non-alcoholic steatohepatitis and preneoplastic lesions develop in the liver of obese and hypertensive rats: Suppressing effects of EGCG on the development of liver lesions. Cancer Lett..

[53] Sakata R., Nakamura T., Torimura T., Ueno T., Sata M. (2013). Green tea with high-density catechins improves liver function and fat infiltration in non-alcoholic fatty liver disease (NAFLD) patients: A double-blind placebo-controlled study. Int. J. Mol. Med..

[54] Darvesh A.S., Bishayee A. (2013). Chemopreventive and therapeutic potential of tea polyphenols in hepatocellular cancer. Nutr. Cancer.

[55] Kuo P.L., Lin C.C. (2003). Green tea constituent (−)-epigallocatechin-3-gallate inhibits HepG2 cell proliferation and induces apoptosis through p53-dependent and Fas-mediated pathways. J. Biomed. Sci..

[56] Nishikawa T., Nakajima T., Moriguchi M., Jo M., Sekoguchi S., Ishii M., Takashima H., Katagishi T., Kimura H., Minami M. (2006). A green tea polyphenol, epigalocatechin-3-gallate, induces apoptosis of human hepatocellular carcinoma, possibly through inhibition of Bcl-2 family proteins. J. Hepatol..

[57] Lin S.C., Li W.C., Shih J.W., Hong K.F., Pan Y.R., Lin J.J. (2006). The tea polyphenols EGCG and EGC repress mRNA expression of human telomerase reverse transcriptase (hTERT) in carcinoma cells. Cancer Lett..

[58] Shimizu M., Shirakami Y., Sakai H., Tatebe H., Nakagawa T., Hara Y., Weinstein I.B., Moriwaki H. (2008). EGCG inhibits activation of the insulin-like growth factor (IGF)/IGF-1 receptor axis in human hepatocellular carcinoma cells. Cancer Lett..

[59] Shirakami Y., Shimizu M., Adachi S., Sakai H., Nakagawa T., Yasuda Y., Tsurumi H., Hara Y., Moriwaki H. (2009). (−)-Epigallocatechin gallate suppresses the growth of human hepatocellular carcinoma cells by inhibiting activation of the vascular endothelial growth factor-vascular endothelial growth factor receptor axis. Cancer Sci..

[60] Sakata R., Ueno T., Nakamura T., Sakamoto M., Torimura T., Sata M. (2004). Green tea polyphenol epigallocatechin-3-gallate inhibits platelet-derived growth factor-induced proliferation of human hepatic stellate cell line LI90. J. Hepatol..

[61] Adachi S., Nagao T., Ingolfsson H.I., Maxfield F.R., Andersen O.S., Kopelovich L., Weinstein I.B. (2007). The inhibitory effect of (−)-epigallocatechin gallate on activation of the epidermal growth factor receptor is associated with altered lipid order in HT29 colon cancer cells. Cancer Res..

[62] Adachi S., Nagao T., To S., Joe A.K., Shimizu M., Matsushima-Nishiwaki R., Kozawa O., Moriwaki H., Maxfield F.R., Weinstein I.B. (2008). (−)-Epigallocatechin gallate causes internalization of the epidermal growth factor receptor in human colon cancer cells. Carcinogenesis.

[63] Yasuda Y., Shimizu M., Sakai H., Iwasa J., Kubota M., Adachi S., Osawa Y., Tsurumi H., Hara Y., Moriwaki H. (2009). (−)-Epigallocatechin gallate prevents carbon tetrachloride-induced rat hepatic fibrosis by inhibiting the expression of the PDGFRβ and IGF-1R. Chem. Biol. Interact..

[64] Tamura K., Nakae D., Horiguchi K., Akai H., Kobayashi Y., Satoh H., Tsujiuchi T., Denda A., Konishi Y. (1997). Inhibition by green tea extract of diethylnitrosamine-initiated but not choline-deficient, l-amino acid-defined diet-associated development of putative preneoplastic, glutathione *S*-transferase placental form-positive lesions in rat liver. Jpn. J. Cancer Res..

[65] Nishida H., Omori M., Fukutomi Y., Ninomiya M., Nishiwaki S., Suganuma M., Moriwaki H., Muto Y. (1994). Inhibitory effects of (−)-epigallocatechin gallate on spontaneous hepatoma in C3H/HeNCrj mice and human hepatoma-derived PLC/PRF/5 cells. Jpn. J. Cancer Res..

[66] Shimizu M., Fukutomi Y., Ninomiya M., Nagura K., Kato T., Araki H., Suganuma M., Fujiki H., Moriwaki H. (2008). Green tea extracts for the prevention of metachronous colorectal adenomas: A pilot study. Cancer Epidemiol. Biomark. Prev..

[67] Bettuzzi S., Brausi M., Rizzi F., Castagnetti G., Peracchia G., Corti A. (2006). Chemoprevention of human prostate cancer by oral administration of green tea catechins in volunteers with high-grade prostate intraepithelial neoplasia: A preliminary report from a one-year proof-of-principle study. Cancer Res..

[68] Li N., Sun Z., Han C., Chen J. (1999). The chemopreventive effects of tea on human oral precancerous mucosa lesions. Proc. Soc. Exp. Biol. Med..

[69] Clark J., You M. (2006). Chemoprevention of lung cancer by tea. Mol. Nutr. Food Res..

[70] Fujiki H., Suganuma M., Imai K., Nakachi K. (2002). Green tea: Cancer preventive beverage and/or drug. Cancer Lett..

[71] Suganuma M., Okabe S., Kai Y., Sueoka N., Sueoka E., Fujiki H. (1999). Synergistic effects of (−)-epigallocatechin gallate with (−)-epicatechin, sulindac, or tamoxifen on cancer-preventive activity in the human lung cancer cell line PC-9. Cancer Res..

[72] Shimizu M., Deguchi A., Lim J.T., Moriwaki H., Kopelovich L., Weinstein I.B. (2005). (−)-Epigallocatechin gallate and polyphenon E inhibit growth and activation of the epidermal growth factor receptor and human epidermal growth factor receptor-2 signaling pathways in human colon cancer cells. Clin. Cancer Res..

[73] Hirose M., Mizoguchi Y., Yaono M., Tanaka H., Yamaguchi T., Shirai T. (1997). Effects of green tea catechins on the progression or late promotion stage of mammary gland carcinogenesis in female Sprague-Dawley rats pretreated with 7,12-dimethylbenz(a)anthracene. Cancer Lett..

[74] Chow H.H., Cai Y., Alberts D.S., Hakim I., Dorr R., Shahi F., Crowell J.A., Yang C.S., Hara Y. (2001). Phase I pharmacokinetic study of tea polyphenols following single-dose administration of epigallocatechin gallate and polyphenon E. Cancer Epidemiol. Biomark. Prev..

[75] Chow H.H., Cai Y., Hakim I.A., Crowell J.A., Shahi F., Brooks C.A., Dorr R.T., Hara Y., Alberts D.S. (2003). Pharmacokinetics and safety of green tea polyphenols after multiple-dose administration of epigallocatechin gallate and polyphenon E in healthy individuals. Clin. Cancer Res..

[76] Mazzanti G., Menniti-Ippolito F., Moro P.A., Cassetti F., Raschetti R., Santuccio C., Mastrangelo S. (2009). Hepatotoxicity from green tea: A review of the literature and two unpublished cases. Eur. J. Clin. Pharmacol..

[77] Jimenez-Saenz M., Martinez-Sanchez Mdel C. (2006). Acute hepatitis associated with the use of green tea infusions. J. Hepatol..

[78] Teschke R., Zhang L., Melzer L., Schulze J., Eickhoff A. (2014). Green tea extract and the risk of drug-induced liver injury. Expert Opin. Drug Metab. Toxicol..

[79] Luo H., Tang L., Tang M., Billam M., Huang T., Yu J., Wei Z., Liang Y., Wang K., Zhang Z.Q. (2006). Phase IIa chemoprevention trial of green tea polyphenols in high-risk individuals of liver cancer: Modulation of urinary excretion of green tea polyphenols and 8-hydroxydeoxyguanosine. Carcinogenesis.

[80] Jin X., Zheng R.H., Li Y.M. (2008). Green tea consumption and liver disease: A systematic review. Liver Int..

[81] McLarty J., Bigelow R.L., Smith M., Elmajian D., Ankem M., Cardelli J.A. (2009). Tea polyphenols decrease serum levels of prostate-specific antigen, hepatocyte growth factor, and vascular endothelial growth factor in prostate cancer patients and inhibit production of hepatocyte growth factor and vascular endothelial growth factor *in vitro*. Cancer Prev. Res. (Phila).

[82] Frezza E.E., Wachtel M.S., Chiriva-Internati M. (2006). Influence of obesity on the risk of developing colon cancer. Gut.

[83] Giovannucci E., Michaud D. (2007). The role of obesity and related metabolic disturbances in cancers of the colon, prostate, and pancreas. Gastroenterology.

[84] Shimizu M., Shirakami Y., Iwasa J., Shiraki M., Yasuda Y., Hata K., Hirose Y., Tsurumi H., Tanaka T., Moriwaki H. (2009). Supplementation with branched-chain amino acids inhibits azoxymethane-induced colonic preneoplastic lesions in male C57BL/KsJ-*db*/*db* mice. Clin. Cancer Res..

[85] Kubota M., Shimizu M., Sakai H., Yasuda Y., Terakura D., Baba A., Ohno T., Tsurumi H., Tanaka T., Moriwaki H. (2012). Preventive effects of curcumin on the development of azoxymethane-induced colonic preneoplastic lesions in male C57BL/KsJ-*db*/*db* obese mice. Nutr. Cancer.

[86] Shirakami Y., Shimizu M., Kubota M., Araki H., Tanaka T., Moriwaki H., Seishima M. (2014). Chemoprevention of colorectal cancer by targeting obesity-related metabolic abnormalities. World J. Gastroenterol..

